# Heat stress memory differentially regulates the expression of nitrogen transporter genes in the filamentous red alga ‘*Bangia*’ sp. ESS1

**DOI:** 10.3389/fpls.2024.1331496

**Published:** 2024-02-05

**Authors:** Natsumi Sato, Ho Viet Khoa, Koji Mikami

**Affiliations:** ^1^School of Food Industrial Sciences, Miyagi University, Sendai, Japan; ^2^Graduate School of Fisheries Sciences, Hokkaido University, Hakodate, Japan

**Keywords:** heat stress, stress memory, hydrogen peroxide, nitrogen transporter, gene expression, red alga

## Abstract

**Introduction:**

To withstand high temperatures that would be lethal to a plant in the naïve state, land plants must establish heat stress memory. The acquisition of heat stress tolerance via heat stress memory in algae has only been observed in the red alga ‘*Bangia*’ sp. ESS1.

**Methods:**

In this study, we further evaluated the intrinsic ability of this alga to establish heat stress memory by monitoring hydrogen peroxide (H_2_O_2_) production and examining the relationship between heat stress memory and the expression of genes encoding nitrogen transporters, since heat stress generally reduces nitrogen absorption. Next, genes encoding nitrogen transporters were selected from our unpublished transcriptome data of ‘*Bangia*’ sp. ESS1.

**Results:**

We observed a reduction in H_2_O_2_ content when heat stress memory was established in the alga. In addition, six ammonium transporter genes, a single-copy nitrate transporter gene and two urea transporter genes were identified. Two of these nitrogen transporter genes were induced by heat stress but not by heat stress memory, two genes showed heat stress memory–dependent expression, and one gene was induced by both treatments. Heat stress memory therefore differentially regulated the expression of the nitrogen transporter genes by reducing heat stress–inducible gene expression and inducing heat stress memory–dependent gene expression.

**Discussion:**

These findings point to the functional diversity of nitrogen transporter genes, which play different roles under various heat stress conditions. The characteristic effects of heat stress memory on the expression of individual nitrogen transporter genes might represent an indispensable strategy for reducing the threshold of sensitivity to recurrent high-temperature conditions and for maintaining nitrogen absorption under such conditions in ‘*Bangia*’ sp. ESS1.

## 1 Introduction

Acclimation to changes in environmental conditions is crucial for the survival of plants, as plants cannot move away from abiotic and biotic stresses. Recent studies have demonstrated that plants “memorize” the experience of exposure to non-lethal environmental changes, allowing them to acquire tolerance to subsequent exposure to environmental changes that would normally be lethal (Sharma et al., 2022; Kambona et al., 2023). Indeed, plants withstand extremely high temperatures, which are lethal to plants in the native state, following growth at optimal temperatures for several days after exposure to non-lethal high temperatures (Yamaguchi, 2021a; Yamaguchi et al., 2021b; Ramakrishnan et al., 2022; Charng et al., 2023). The establishment of stress memory is therefore a critical strategy for survival under recurrent environmental changes.

Members of Bangiales, an order of multicellular red algae with a filamentous or foliose shape (Sutherland et al., 2011; Mikami and Takahashi, 2023), grow abundantly in intertidal regions, where temperatures often fluctuate widely (Karsten and West, 2000; Chen et al., 2022). Recent transcriptome analyses indicated that foliose Bangiales algae respond to heat stress via the heat-inducible expression of genes encoding heat shock proteins (HSPs) (Kim et al., 2011; Sun et al., 2015; Wang et al., 2018; Jin et al., 2020; Chang et al., 2021; Yu et al., 2021; Gao et al., 2022; Wi et al., 2023). In the foliose red alga *Pyropia yezoensis*, membrane fluidization at high temperatures triggers the heat stress–inducible expression of *HSP70* and *multiprotein bridging factor 1* (*MBF1*) genes, whereas heat stress–inducible expression of *high temperature response 2* (*HTR2*) and *HTR2-like* (*HTR2L*) genes occurs independently of membrane fluidization (Khoa and Mikami, 2022; Mikami and Khoa, 2023). Thus, Bangiales algae perceive and respond to high temperature via heat stress–inducible gene expression, and the responses are regulated by multiple intracellular signal transduction pathways (Khoa and Mikami, 2022). However, little is known about heat stress tolerance and memory in foliose Bangiales.

By contrast, the existence of heat stress memory has been confirmed in filamentous Bangiales. For example, the intrinsic ability to acquire heat stress tolerance by establishing heat stress memory was observed in ‘*Bangia*’ sp. ESS1 of the ‘*Bangia*’ 2 group (Kishimoto et al., 2019; Li et al., 2019a; Khoa et al., 2021). However, *Bangia atropurpurea* acquires heat stress tolerance but is unable to remember heat stress, and ‘*Bangia*’ sp. ESS2 of the ‘*Bangia*’ 3 group cannot acquire heat stress tolerance or establish stress memory (Khoa et al., 2021). These findings indicate that physiological responses to heat stress vary among ‘*Bangia’* species, although it is unknown whether the presence of stress memory depends on phylogenetic classification (genus *Bangia* and ‘*Bangia*’ groups 1, 2, and 3; Sutherland et al., 2011; Mikami and Takahashi, 2023) or the characteristics of individual species.

High temperature usually inhibits the growth of macroalgae (Endo et al., 2020; Fernández et al., 2020; Wu et al., 2022). In addition, high temperature reduces nitrogen uptake and nitrogen content in these algae (Gerard, 1997; Hay et al., 2010; Gouvêa et al., 2017). Thus, growth retardation and reduced nitrogen accumulation are tightly correlated, which is consistent with the finding that nitrogen is an indispensable macronutrient for plant growth in most terrestrial and aquatic ecosystems (Edward and Richard, 2001; Witte, 2011). Indeed, nitrogen fertilization promotes growth under heat stress conditions in brown, green, and red algae (Wang et al., 2014; Gouvêa et al., 2017; Fernández et al., 2020; Wu et al., 2022; Wang et al., 2023). Moreover, high temperature stimulates nitrogen accumulation in meristems of the brown alga *Eisenia bicyclis* and the green alga *Ulva prolifera* (Endo et al., 2020; Sato et al., 2021). These findings suggest that the effects of high-temperature stress on nitrogen uptake differ among phyla, genera, and/or species of algae, but how heat stress influences the uptake and accumulation of nitrogen sources in algae remains to be elucidated.

Nitrogen sources comprise organic forms such as urea, amino acids, free peptides, and proteins, as well as the inorganic forms nitrate (NO_3_^−^), nitrite (NO_2_^−^), and ammonium (NH_4_^+^), all of which are major nitrogen sources in soil and seawater (Okumoto and Versaw, 2017). The uptake of these nitrogen sources into cells is mediated by transporters that differentially recognize inorganic or organic nitrogen sources (Gazzarrini et al., 1999; Ludewig et al., 2007, Pinton et al., 2016). For example, the influx of extracellular NH_4_^+^ into cells is mediated by ammonium transporters (AMTs; D’Apuzzo et al., 2004), whereas urea uptake into cells occurs via urea transporters (DUR3s; Wang et al., 2008a). In addition, NO_3_^−^ is imported into cells by nitrate transporters (NRTs), which form a large family with many members and distinct functions in plants (Bai et al., 2013). Thus, AMT, DUR3, and NRT are critical factors in the influx of nitrogen sources.

Despite our increasing knowledge about the presence of nitrogen transporters in terrestrial plants and algae, it is unclear whether there is a relationship between nitrogen uptake and the acquisition of stress tolerance under high-temperature conditions in Bangiales. Because the physiology of the heat stress response in ‘*Bangia*’ species has been well studied (Notoya and Iijima, 2003; Wang et al., 2008b; Mikami and Kishimoto, 2018; Kishimoto et al., 2019; Li et al., 2019a; Khoa et al., 2021), perhaps ‘*Bangia*’ sp. ESS1, which memorizes heat stress to acquire heat stress tolerance (Kishimoto et al., 2019; Khoa et al., 2021), could serve as a model system for investigating the expression profiles of nitrogen transporter genes under heat stress in red algae.

In this study, the intrinsic ability of ‘*Bangia*’ sp. ESS1 to establish heat stress memory was confirmed by monitoring production of hydrogen peroxide (H_2_O_2_). Then, the ‘*Bangia*’ sp. ESS1 genes encoding nitrogen transporters such as AMT, NRT, and DUR3 were identified and their expression profiles were analyzed during the acquisition of heat stress tolerance and the establishment of heat stress memory. Our findings reveal novel aspects of the relationship between heat stress memory and nitrogen uptake under heat stress conditions in Bangiales.

## 2 Materials and methods

### 2.1 Algal materials and culture conditions for maintenance

Filamentous gametophytic thalli of ‘*Bangia*’ sp. ESS1 were harvested in Esashi, Hokkaido, Japan on May 17, 2010 (Hirata et al., 2011) and phylogenetically classified as a member of ‘*Bangia*’ group 2 (Li et al., 2019a). The alga was maintained clonally as an experimental line in our laboratory in sterilized artificial seawater as described by Li et al. (2019b) under 60–70 μmol photons m^−2^ s^−1^ light with a short-day photoperiod (10 h light/14 h dark) at 15°C and aerated with air filtered through a 0.22-μm filter (Whatman, Maidstone, UK). The culture medium was changed weekly.

### 2.2 Stress treatment of algal materials

**Figure 1:**
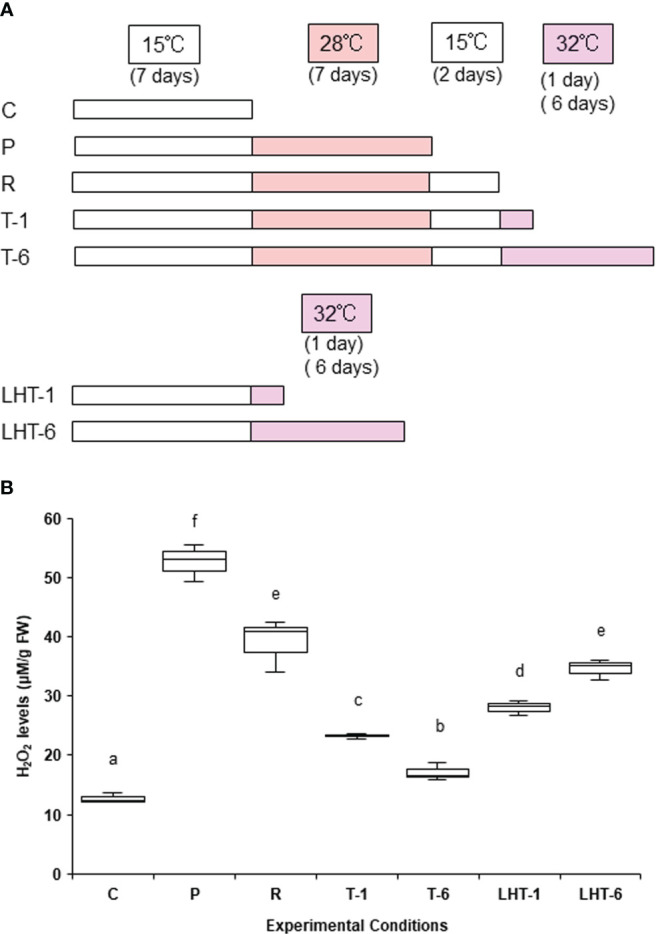
Effects of heat stress memory on hydrogen peroxide (H_2_O_2_) production in ‘*Bangia*’ sp. ESS1. **(A)** Schematic representation of the experimental conditions, including priming, recovery, and triggering, used to assess the biological significance of heat stress memory in this study. Seven conditions were employed: control, growth at 15°C (C); priming, incubation at 28°C for 7 days after growth at 15°C (P); recovery from 28°C treatment by incubation at 15°C for 2 days (R); triggering, 32°C treatment for 1 or 6 days (T-1 and T-6, respectively); and direct transfer to a lethal high temperature 32°C for 1 or 6 days (LHT-1 and LHT-6, respectively). **(B)** Quantitative analysis of H_2_O_2_ production under various heat stress conditions. H_2_O_2_ contents in algae treated with the experimental conditions indicated in **(A)** were quantified; mean values ± SD per 0.2 g sample fresh weight were calculated from three independent experiments. Different letters denote statistically significant differences (*p* < 0.05), as determined by one-way ANOVA.

Samples (0.05 g fresh weight) of thalli cultured under aeration at 15°C were incubated without agitation in dishes (*Azunoru dish, 90 mm diameter × 20 mm height; As One Co., Ltd., Osaka, Japan*) containing 50 ml of seawater at 15°C for 7 days to adapt to changes in culture conditions. We previously demonstrated that ‘*Bangia*’ sp. ESS1 acquires heat stress tolerance by establishing heat stress memory, which is mostly maintained for 2 days after recovery from a 7-day non-lethal stress (Kishimoto et al., 2019). Thus, the samples were then subjected to one of six different stress treatments (**Figure 1A**): (1) 28°C for 7 days (priming, P); (2) 32°C for 1 day (lethal high temperature-1, LHT-1); (3) 32°C for 6 days (lethal high temperature-6, LHT-6); (4) 28°C for 7 days and 15°C for 2 days (recovery, R); (5) 28°C for 7 days, 15°C for 2 days, and 32°C for 1 day (triggering-1, T-1); and (6) 28°C for 7 days, 15°C for 2 days, and 32°C for 6 days (triggering-6, T-6). The control condition was incubation at 15°C for 7 days. Samples from all stress treatments from three repeated experiments (three samples per treatment) were harvested, frozen in liquid nitrogen, and stored at −80°C prior to H_2_O_2_ quantification and gene expression analysis.

### 2.3 Quantitative analysis of hydrogen peroxide production

The H_2_O_2_ contents of samples treated with the various stress conditions described above were measured as described by Kumar et al. (2011) with slight modifications. Initially, 200-mg (fresh weight) algal samples were extracted with 400 μl of 50 mM Na-acetate buffer (pH 6.5) (1:2, w/v). A 100-μl aliquot of each extract was incubated in a reaction mixture consisting of 50 mM Na-acetate buffer, 1 mM 4-aminoantipyrine, 1 mM 2,4-dichlorophenol, 50 mM MnCl_2_, and 0.2 mM NADH for 24 h. Finally, the oxidation of aminoantipyrine was measured as the absorbance of the reaction mixture at 510 nm, which was compared to a previously prepared standard curve to determine the H_2_O_2_ concentration in each sample.

### 2.4 Identification of nitrogen transporter genes

Unigenes annotated as putative nitrogen transporter genes were selected from our unpublished transcriptome data from ‘*Bangia*’ sp. ESS1, and their identity was confirmed by comparing their predicted amino acid sequences with those of known nitrogen transporters by a BLAST search (https://blast.ncbi.nlm.nih.gov/Blast.cgi) after identifying full-length open reading frames (ORFs) with the ORF finder (https://www.ncbi.nlm.nih.gov/orffinder/). Complete mRNA sequences from these genes have been deposited at DDBJ/EMBL/GenBank; their accession numbers are listed next to the species names in the phylogenetic trees.

### 2.5 Phylogenetic analyses of nitrogen transporters in plants and algae

To examine the evolutionary relationships of the ‘*Bangia*’ sp. ESS1 nitrogen transporters with those from other plants and algae, neighbor-joining phylogenetic trees were constructed with MEGA 7 software (https://www.megasoftware.net) using ClustalW to align the sequences of orthologs from other organisms. The amino acid sequences of AMTs, NRTs, and DUR3s used for the phylogenetic analysis were obtained from the GenBank, genome, and EST databases. The accession numbers and IDs of these sequences are indicated next to the species names in the phylogenetic trees.

### 2.6 Total RNA extraction and cDNA synthesis

Total RNA was extracted using a FavorPrep Plant Total RNA Mini Kit (FAVORGEN, Ping Tung, Taiwan) and treated with DNase I to remove any genomic DNA contamination using a TURBO DNA-free kit (Thermo Fisher Scientific, Waltham, MA, USA). Total RNA samples (300 ng) with A260/A280 ratios ranging from 1.9 to 2.1 were used to synthesize first-strand complementary DNA (cDNA) with a PrimeScript 1st strand cDNA Synthesis Kit (Takara Bio, Kusatsu, Japan). The thermal cycling parameters consisted of an initial denaturation step at 98°C for 30 s, followed by 30 cycles of 98°C for 10 s, 60°C for 30 s, and 72°C for 20 s, and a final extension step at 72°C for 5 min.

### 2.7 Reverse-transcription PCR for quantitative gene expression analysis

Primers for quantitative reverse-transcription PCR (qRT-PCR) were designed using Primer Premier 5 (http://www.premierbiosoft.com) as shown in **Supplementary Table S1**. To confirm the sizes of the amplified products and the suitability of the primers, a mixture of three cDNA samples was used with all primer sets for PCR with Phusion high-fidelity DNA polymerase and GC buffer (New England BioLabs, MA, USA) according to the manufacturer’s instructions. Primer sets that amplified DNA bands of the expected sizes, as checked by agarose gel electrophoresis, were employed for qPCR. qPCR was performed in a total volume of 20 μl containing 10 μl of 2× SYBR Premix Ex Taq GC, 0.4 μl of ROX Reference Dye, 2 μl of cDNA template, and 0.4 μl (10 μM) of each primer using a SYBR Premix Ex Taq GC kit (Takara Bio, Kusatsu, Japan). The thermal cycling parameters for the AriaMX (3000P) real-time PCR system (Agilent Technologies, CA, USA) consisted of 95°C for 3 min and 40 cycles of 95°C for 5 s and 60°C for 20 s. A dissociation curve was generated to confirm the specificity of amplification at 95°C for 1 min, 55°C for 30 s, and 95°C for 30 s.

### 2.8 Statistical analysis

Values are means ± SD from triplicate experiments. One-way ANOVA followed by a Tukey-Kramer test was used for multiple comparisons, and significant differences were determined using a cutoff value of *p* < 0.05.

## 3 Results

### 3.1 Heat stress memory reduces hydrogen peroxide production

Since one of the earliest physiological responses to heat stress in a variety of organisms is the production of reactive oxygen species (ROS) (Sachdev et al., 2021; Fortunato et al., 2023), we investigated the heat responses of ‘*Bangia*’ sp. ESS1 by monitoring its heat stress–dependent production of hydrogen peroxide (H_2_O_2_). When ‘*Bangia*’ sp. ESS1 was exposed to a non-lethal high temperature (28°) for 7 days (priming [P] in **Figure 1A**), H_2_O_2_ highly accumulated (**Figure 1B**), indicating that exposure to heat stress promotes H_2_O_2_ production in this alga as in other organisms.

We then quantified H_2_O_2_ contents during the establishment of heat stress memory to explore the relationship between heat stress memory and H_2_O_2_ production. When ‘*Bangia*’ sp. ESS1 was subjected to a 7-day heat stress at 28°C followed by recovery at 15°C for 2 days (recovery [R] in **Figure 1A**), H_2_O_2_ was still elevated at the end of the treatment (**Figure 1B**), suggesting that the physiological status induced by heat stress was maintained under the non-stressful temperature 15°C. Since stress memory enables survival under naturally lethal high temperatures (Kishimoto et al., 2019), we examined the effects of 32°C treatments on the H_2_O_2_ contents of algal samples. When ‘*Bangia*’ sp. ESS1 was incubated at 32°C for 1 day after a 28°C–15°C treatment (T-1 in **Figure 1A**), the H_2_O_2_ contents decreased, and the decrease was even greater after 6 days at 32°C (T-6 in **Figures 1A, B**). In contrast, the direct transfer of the alga from 15°C to 32°C for 1 or 6 days (LHT-1 and LHT-6 in **Figure 1A**) increased H_2_O_2_ contents (**Figure 1B**). These findings indicate that the establishment of heat stress memory decreases the heat stress–dependent production of H_2_O_2_ in ‘*Bangia*’ sp. ESS1.

### 3.2 Nitrogen transporter genes in ‘*Bangia*’ sp. ESS1

Based on the functional annotation in our unpublished ‘*Bangia*’ sp. ESS1 transcriptome data, we identified six unigenes (CL2278, CL2683, CL232, CL2570, CL337, and Unigene24217) as candidate *AMT* (*BE1AMT*) genes, one unigene (Unigene22285) as a candidate *NRT* (*BE1NRT*) gene, and two unigenes (CL2421 and Unigene31059) as candidate *DUR3* (*BE1DUR3*) genes in ‘*Bangia*’ sp. ESS1. To explore what type(s) of nitrogen transporters these unigenes encode, we performed phylogenetic analysis using the amino acid sequences of AMTs, NRTs, and DUR3s from ‘*Bangia*’ sp. ESS1, other algae, and various terrestrial plants.

**Figure 2:**
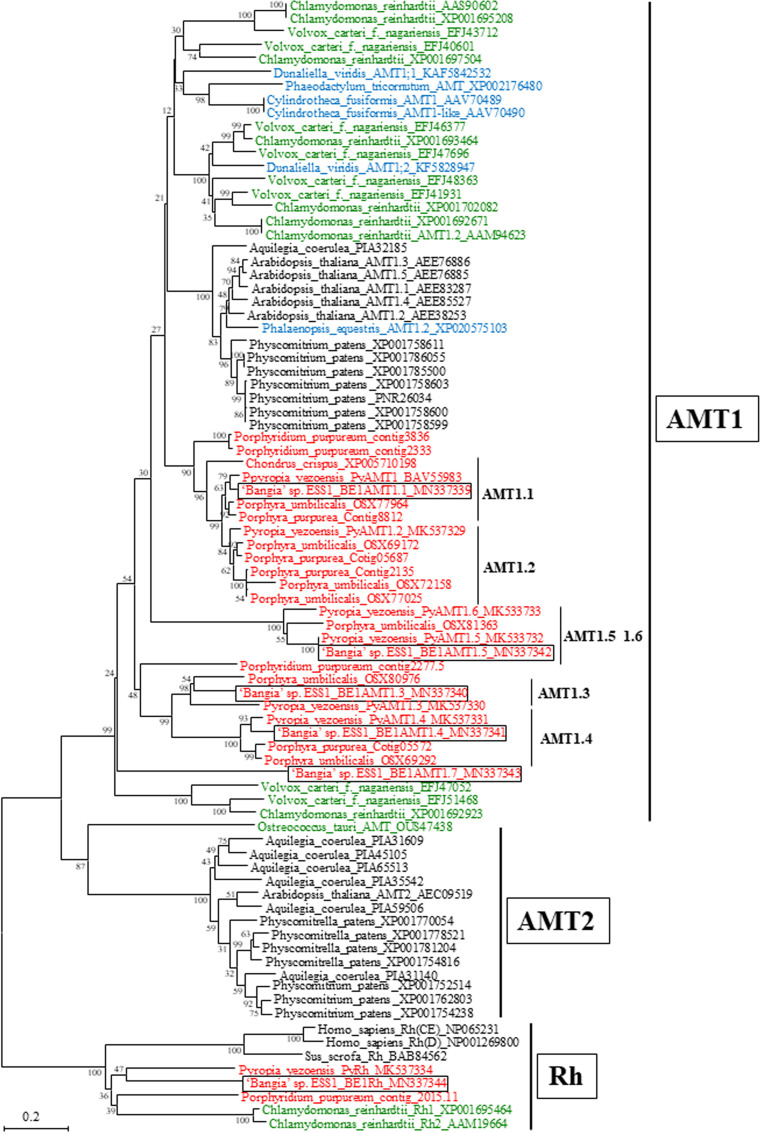
Neighbor-joining-based phylogenetic tree of AMTs from ‘*Bangia*’ sp. ESS1 compared with those of terrestrial plants and other algae. Boxes indicate BE1AMTs and BE1Rh from ‘*Bangia*’ sp. ESS1. AMTs from Rhodophyta, Chlorophyta, and Ochrophyta are highlighted by red, green, and blue font, respectively. The bootstrap values from 1000 replicates are indicated at the nodes of the tree. The DDBJ/EMBL/GenBank accession numbers of the AMTs and Rhs used in the phylogenetic analysis are shown next to the species names. Bar, 0.2 substitutions per site.

As shown in **Figure 2**, five of the six candidate ‘*Bangia*’ sp. ESS1 AMTs were placed in the plant AMT1 subfamily clade. Although AMTs in terrestrial plants are encoded by a multigene family comprising the AMT1 and AMT2 subfamilies (Couturier et al., 2007; Wittgenstein et al., 2014), no AMT2 subfamily members were identified in ‘*Bangia*’ sp. ESS1, which is consistent with our previous finding in *P. yezoensis* (Li et al., 2019b). Accordingly, these unigenes were designated *BE1AMT1.1* (CL2278), *BE1AMT1.3* (CL232), *BE1AMT1.4* (CL2570), *BE1AMT1.5* (CL2683), and *BE1AMT1.7* (CL337). The names of these unigenes were derived from their *AMT1* orthologs in *P. yezoensis* (Kakinuma et al., 2017; Li et al., 2019b), except for *BE1AMT1.7*, whose ortholog was not found in *P. yezoensis*. No orthologs of *PyAMT1.2* or *PyAMT1.6* were found in ‘*Bangia*’ sp. ESS1. Four BE1AMT1 family members diverged into four clades containing counterparts from *P. yezoensis* and *Porphyra umbilicalis* (**Figure 2**). For instance, the clade containing BE1AMT1 also contains PyAMT1, the clade containing BE1AMT1.3 and BE1AMT 1.4 contains PyAMT1.3 and PyAMT1.4, and the clade containing BE1AMT1.5 contains PyAMT1.5. It is notable that five BE1AMT1s commonly contained amino acid residues conserved in AMT signature and ammonium ion binding and transport (**Supplementary Figure 1**), suggesting functionality of these transporter proteins as AMT. These findings point to the conservation of functional diversity among *AMT1* genes in Bangiales.

Unigene24217 falls into the clade containing PyRh (Li et al., 2019b), which is phylogenetically divergent from both AMT1 and AMT2 (**Figure 2**). Indeed, a product of Unigene24217 shares 30.65% identity with PyRh from *P. yezoensis* but only 17.39% identity with BE1AMT1. Thus, Unigene24217 was designated *BE1Rh* (low homology between BE1AMTs and BE1Rh is represented in **Supplementary Figure 1**).

**Figure 3:**
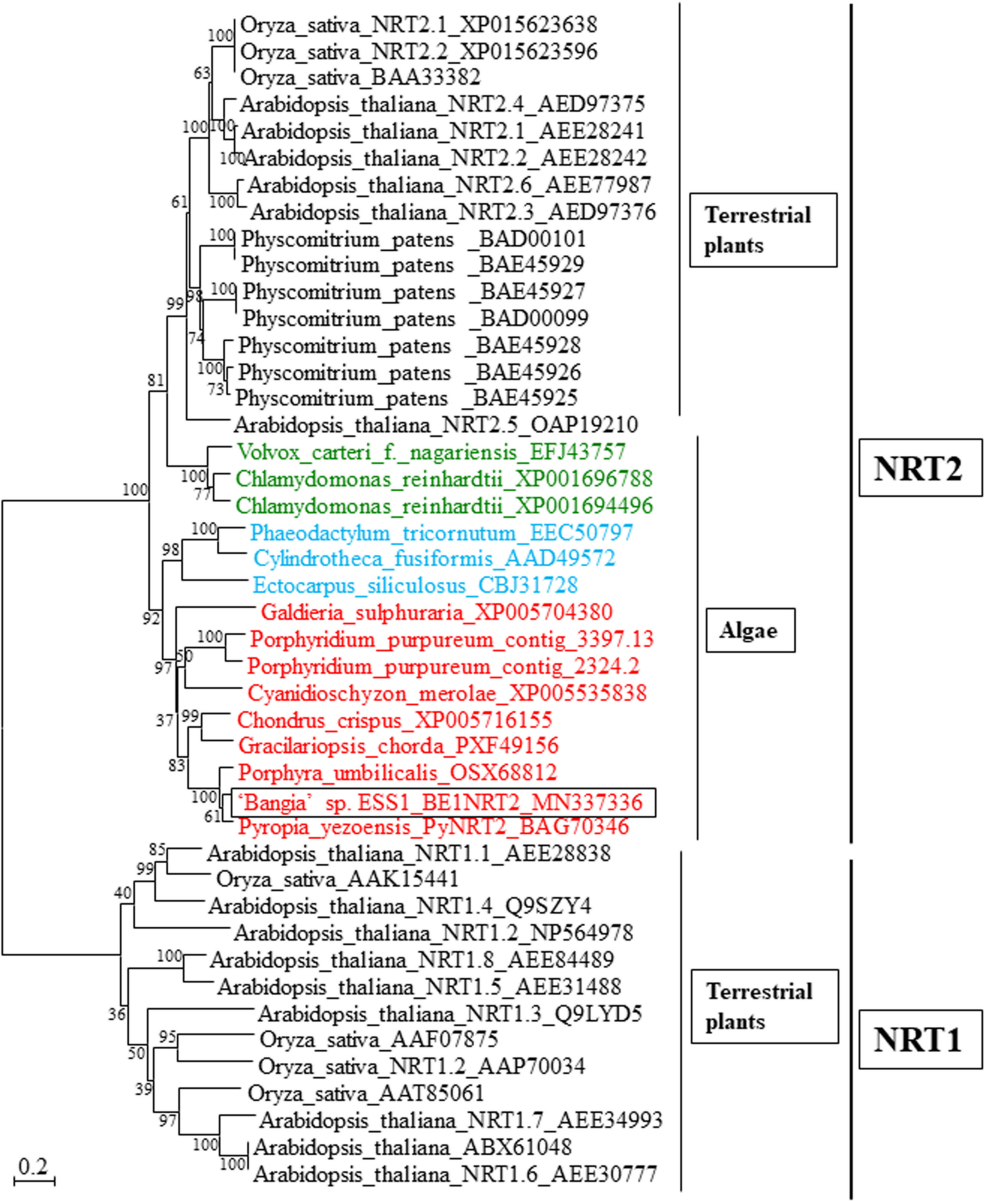
Neighbor-joining-based phylogenetic tree of NRTs from terrestrial plants and algae. The box indicates BE1NRT2 from ‘*Bangia*’ sp. ESS1. NRT2s from Rhodophyta, Chlorophyta, and Ochrophyta are highlighted by red, green, and blue font, respectively. The bootstrap values from 1000 replicate are indicated at the nodes of the tree. The DDBJ/EMBL/GenBank accession numbers of the NRTs used in the phylogenetic analysis are shown next to the species names. Bar, 0.2 substitutions per site.

Unigene22285 formed a multicellular red algal clade with NTR2s from *Chondrus crispus*, *Gracilariopsis chorda*, *Po. umbilicalis*, and *P. yezoensis*, all of which contain a single *NRT2* gene (**Figure 3**). Thus, Unigene22285 was designated *BE1NRT2* that contains NRT2 consensus motif AGWGNLG (**Supplementary Figure 2**). In land plants, three different gene families of nitrate transporters have been identified, NRT1, NRT2, and NRT3 (Plett et al., 2010), all of which symport NO_3_^−^ and protons, with low or high affinity for NO_3_^−^ (Pinton et al., 2016). In addition, the genome of the unicellular green alga *Chlamydomonas reinhardtii* contains six *NRT2* genes (Higuera et al., 2016), and the unicellular red alga *Porphyridium purpureum* contains two *NRT2* genes (**Figure 3**). Therefore, the presence of a single-copy *NRT2* gene (**Figure 2**; Kakinuma et al., 2008; Brawley et al., 2017; Li et al., 2019b) is unique to multicellular Bangiales.

**Figure 4:**
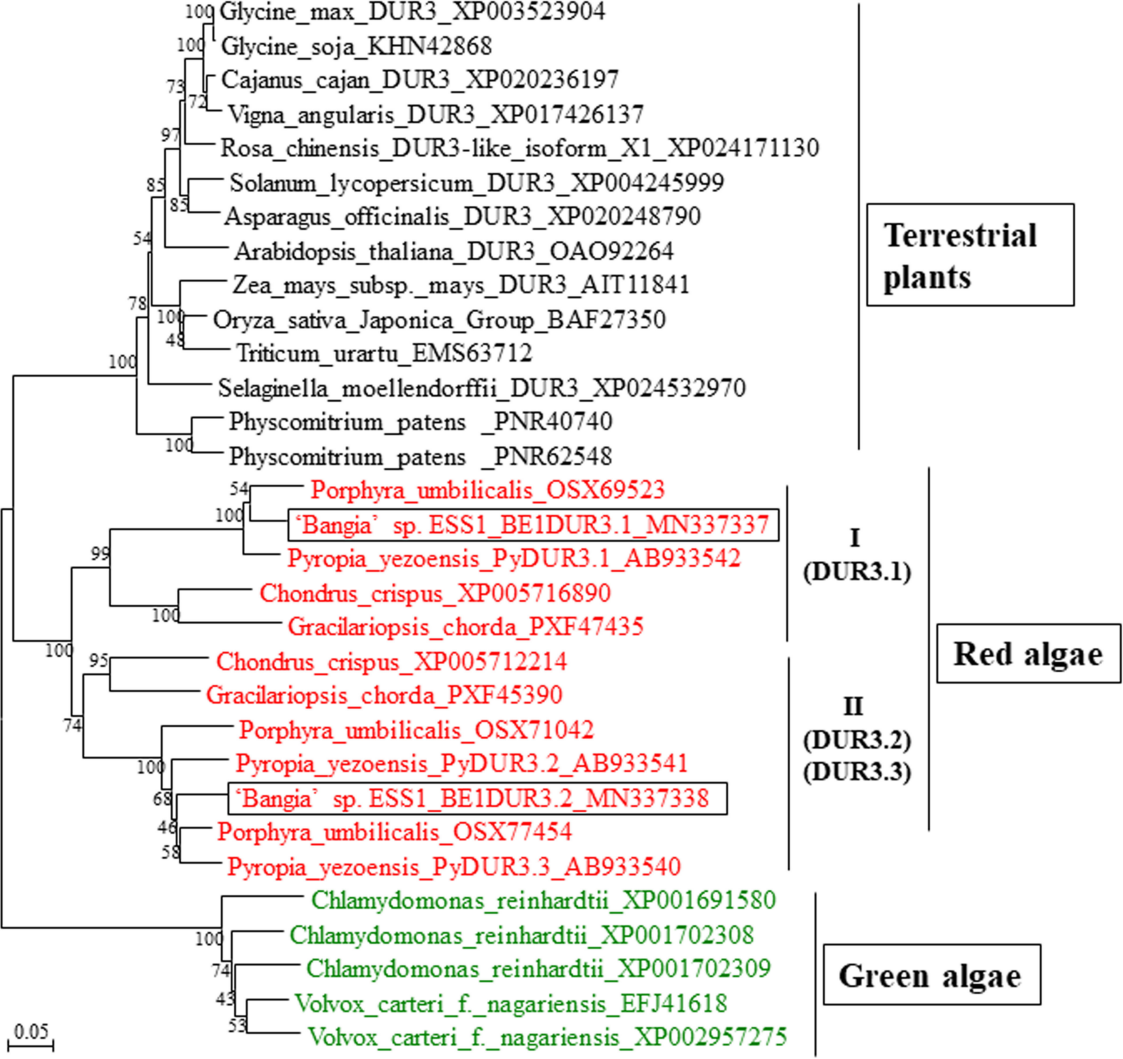
Neighbor-joining-based phylogenetic tree of DUR3s from ‘*Bangia*’ sp. ESS1 compared with those of terrestrial plants and other algae. Boxes indicate BE1DUR3.1 and BE1DUR3.2 from ‘*Bangia*’ sp. ESS1. DUR3s from Rhodophyta and Chlorophyta are highlighted by red and green font, respectively. The bootstrap values from 1000 replicates are indicated at the nodes of the tree. The DDBJ/EMBL/GenBank accession numbers of the DUR3s used in the phylogenetic analysis are shown next to the species names. Bar, 0.05 substitutions per site.

There are two red algal clades (I and II) of DUR3s (**Figure 4**), each of which contains one or two DUR3 family members from other red algae such as *P. yezoensis*, *Po. umbilicalis*, *C. crispus*, and *G. chorda* (Kakinuma et al., 2008; Collén et al., 2013; Kakinuma et al., 2016; Brawley et al., 2017; Kakinuma et al., 2017; Lee et al., 2018). Since CL2421 was included in clade I with PyDUR3.1 and Unigene31059 belongs to clade II with PyDUR3.2 and PyDUR3.3 (**Figure 4**), CL2421 and Unigene31059 were designated *BE1DUR3.1* and *BE1DUR3.2*, respectively. These findings are inconsistent with the previous finding that only a single gene encoding DUR3 is present in the genomes of vascular plants (Kojima et al., 2007; Wang et al., 2012; Liu et al., 2014; Pinton et al., 2016). Amino acid sequence of BE1DUR3s represented high homology to those of DUR3s from *P. yezoensis* and *Po. umbilicalis* (**Supplementary Figure 3**), suggesting functionality of two BE1DUR3s as urea transporters.

### 3.3 Effects of heat stress memory on the expression of ammonium transporter genes

**Figure 5:**
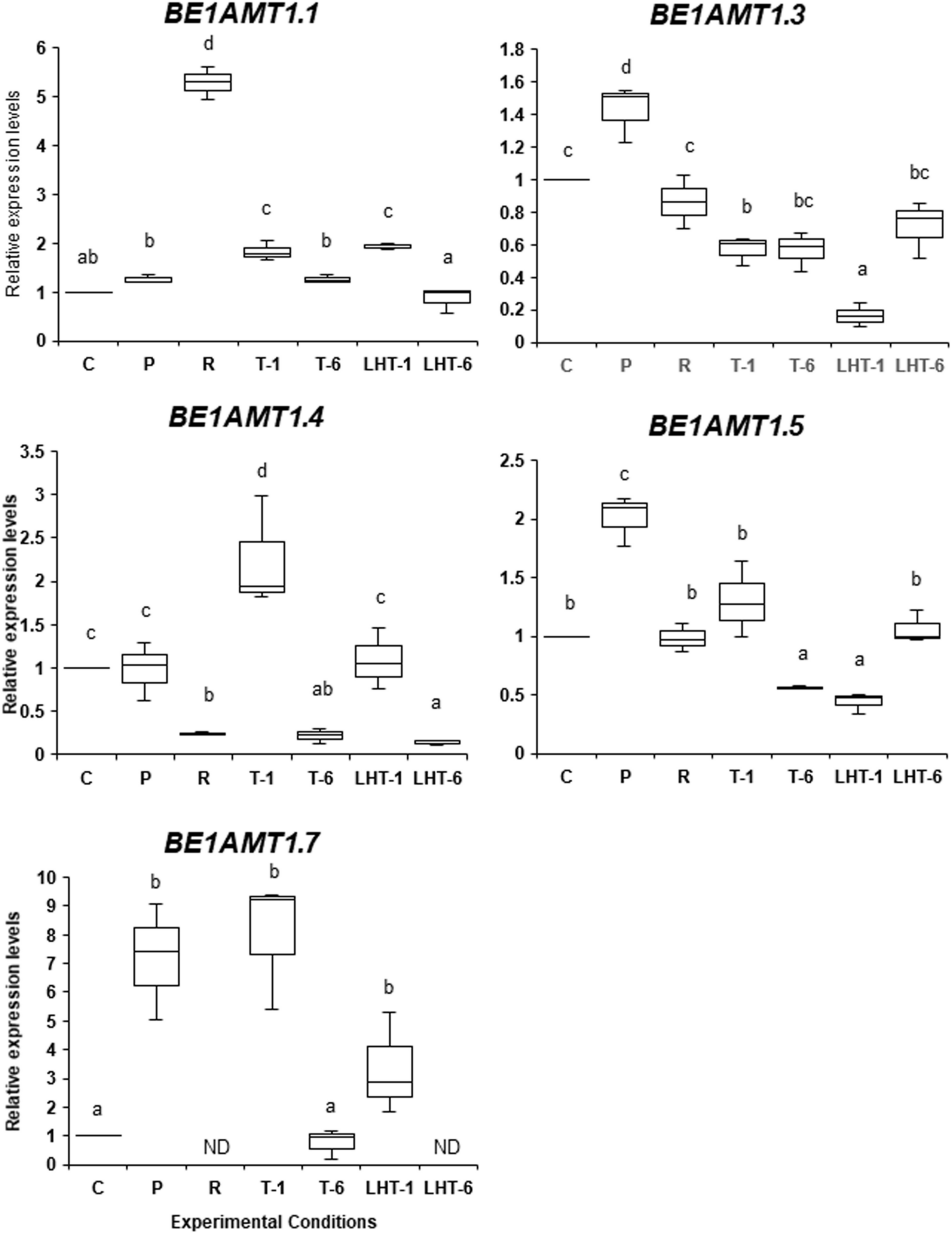
Differences in the expression patterns of the five *BE1AMT1* genes under various heat stress conditions. The expression levels of *BE1AMT1.1*, *BE1AMT1.3*, *BE1AMT1.4*, *BE1AMT1.5*, and *BE1AMT1.7* in algal samples treated with the experimental conditions indicated in **Figure 1A** were measured by qRT-PCR. Relative mRNA levels, which were normalized to the expression of the *Actin* gene as the reference (Li et al., 2019a), are mean fold changes compared to control (C) samples, with error bars representing the standard deviations of triplicate experiments (*n* = 3), each with triple technical replicates for qRT-PCR. ND, not detected. Different letters denote statistically significant differences (*p* < 0.05), as determined by one-way ANOVA.

As shown in **Figure 5**, the expression of *BE1AMT1.3*, *BE1AMT1.5*, and *BE1AMT1.7* was highly induced by 7 days of 28°C treatment, although the expression levels of *BE1AMT1.1* and *BE1AMT1.4* were unchanged. In addition, the recovery treatment (7 day-28°C to 2 day-15°C) reduced the expression levels of all these genes except for *BE1AMT1.1*, whose expression was induced 5-fold by this treatment. The expression profiles of these genes in the triggering experiments (recovery treatment plus 32°C for 1 or 6 days) varied among genes. For example, no response to the lethal temperature was observed for *BE1AMT1.1*, whereas the expression of *BE1AMT1.4* and *BE1AMT1.7* increased by 2 and 8 times, respectively, following 1 day at 32°C and subsequently decreased after 6 days at this temperature. In addition, under the same conditions, *BE1AMT1.5* expression decreased, whereas *BE1AMT1.3* expression was maintained.

When ‘*Bangia*’ sp. ESS1 was incubated at 32°C for 1 or 6 days with no pretreatment (LH-1 and LH-2 in **Figure 1**), the expression patterns of the *BE1AMT1* genes did not strongly vary (**Figure 5**). For instance, *BE1AMT1.1* and *BE1AMT1.7* were induced 2-fold by 1 day of 32°C treatment, whereas 6 days of this treatment completely inhibited their expression. *BE1AMT1.3* and *BE1AMT1.5* expression decreased following 1 day of 32°C treatment and recovered in response to 6 days of treatment. *BE1AMT1.4* expression did not significantly change in response to 1 day of heat treatment and decreased in response to 6 days of heat treatment. A comparison of the gene expression profiles between the triggering treatments and the direct transfer to lethal temperature indicated that stress memory influences the expression of *BE1AMT1.3*, *BE1AMT1.4*, and *BE1AMT1.5*, whereas heat stress alone induces the expression of *BE1AMT1.7*.

### 3.4 Effects of heat stress memory on nitrate transporter gene expression

**Figure 6:**
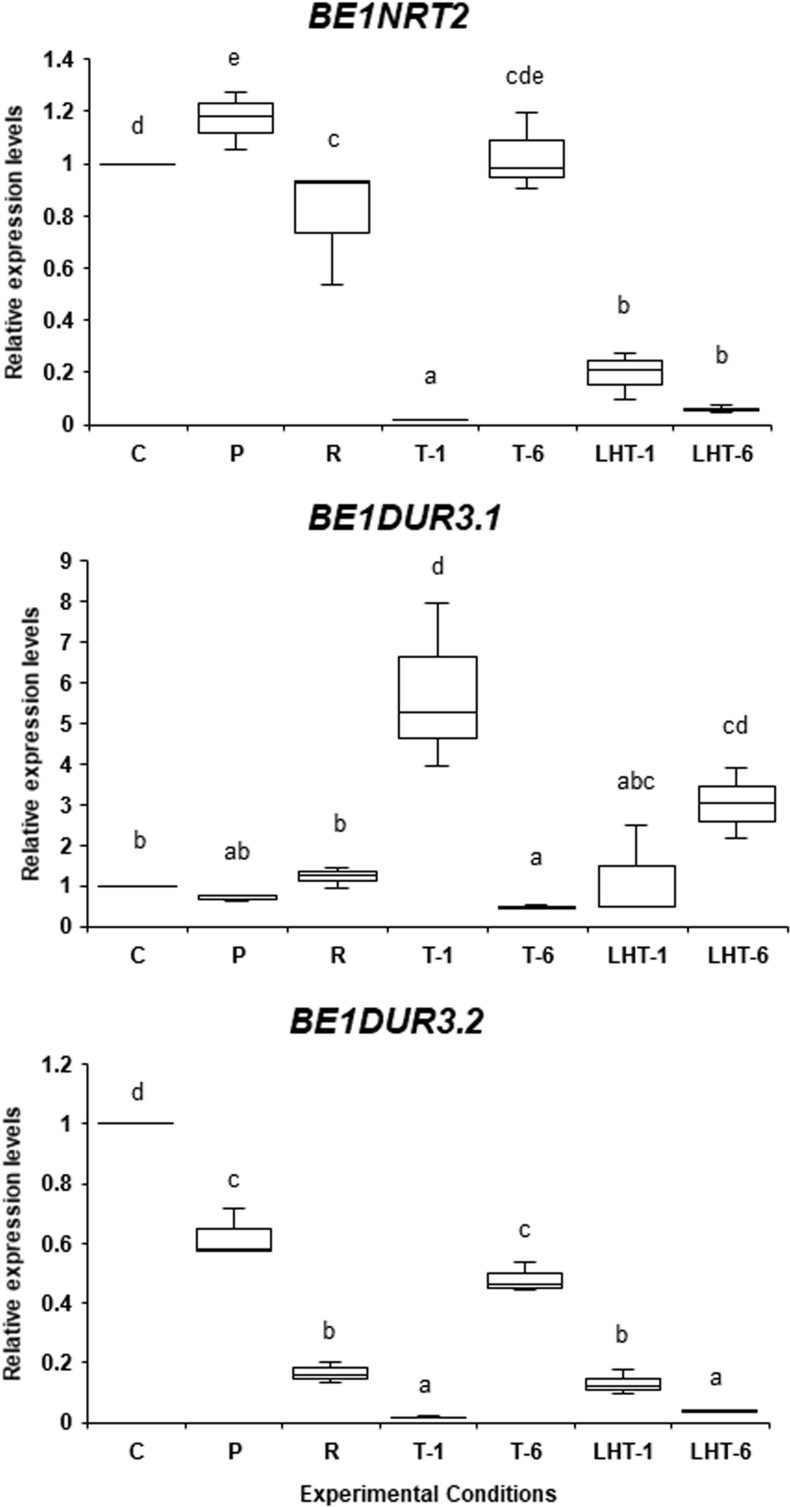
Differences in the expression patterns of *BE1NRT2* and *BE1DUR3* genes under various heat stress conditions. The expression levels of *BE1NRT2*, *BE1DUR3.1*, and *BE1DUR3.2* in algal samples treated with the experimental conditions indicated in **Figure 1A** were examined by qRT-PCR. Relative mRNA levels, which were normalized to the expression of the *Actin* gene as the reference (Li et al., 2019a), are mean fold changes compared to control (C) samples, with error bars representing the standard deviations of triplicate experiments (*n* = 3), each with triple technical replicates for qRT-PCR. Different letters denote statistically significant differences (*p* < 0.05), as determined by one-way ANOVA.

As shown in **Figure 6**, *BE1NRT2* expression slightly increased in response to non-lethal heat stress but strongly decreased after direct transfer to lethal high temperature for 1 or 6 days. Expression of *BE1NRT2* was normal after 6 days of lethal heat in the triggering treatment, but the triggering treatment caused a more extreme decrease in expression after 1 day at 32°C when compared to direct transfer to the lethal temperature. Although this additional reduction in *BE1NRT2* expression by triggering was unexpected, we suggest that this gene is not actively involved in heat stress memory–dependent responses.

### 3.5 Effects of heat stress memory on urea transporter gene expression

The expression profile of *BE1DUR3.1* was similar to that of *BE1AMT1.4*: *BE1DUR3.1* expression was induced 5-fold by a 1-day exposure to 32°C after the triggering treatment and highly decreased in response to a 6-day exposure to 32°C after the triggering treatment (**Figure 6**). Although direct exposure to 32°C for 6 days slightly increased the expression of *BE1DUR3.1* (**Figure 6**), *BE1AMT4* expression did not increase under the same conditions (**Figure 5**). These findings indicate that *BE1DUR1.1* expression is dependent on heat stress memory. Finally, all treatments reduced the expression of *BE1DUR3.2* (**Figure 6**), suggesting that this gene is not involved in the heat stress response in ‘*Bangia*’ sp. ESS1.

## 4 Discussion

Heat stress activates various signal transduction pathways to induce cellular responses to high temperature, including the acquisition of heat stress tolerance and the establishment of heat stress memory (Yamaguchi, 2021a; Yamaguchi, 2021b; Ramakrishnan et al., 2022; Sharma et al., 2022; Charng et al., 2023; Kambona et al., 2023). Although many studies have shed light on these physiological responses in terrestrial plants, our knowledge about tolerance to and memory of heat stress in algae is limited. To date, the presence of an intrinsic ability to establish heat stress memory in algae has only been demonstrated in the red alga ‘*Bangia*’ sp. ESS1 (Kishimoto et al., 2019; Khoa et al., 2021). Since heat stress generally reduces nitrogen uptake and growth in algae (Gerard, 1997; Hay et al., 2010; Gouvêa et al., 2017; Endo et al., 2020; Fernández et al., 2020; Wu et al., 2022), it is important to address whether the establishment of heat stress memory modulates the uptake of nitrogen sources under heat stress conditions in algae. Therefore, in this study, we identified ‘*Bangia*’ sp. ESS1 genes encoding the nitrogen transporters BE1AMT1s, BE1NRT2, and BE1DUR3s and examined the effects of heat stress memory on their expression.

We first evaluated the intrinsic ability to establish heat stress memory in ‘*Bangia*’ sp. ESS1 by monitoring the accumulation of H_2_O_2_ under various heat stress conditions. Although a 7-day incubation at 28°C prompted the production of H_2_O_2_, heat stress memory reduced the amount of H_2_O_2_ produced at 32°C. These results indicate that heat stress memory reduces the sensitivity of the red alga to lethal high temperatures; this results in a reduction in H_2_O_2_ content at 32°C by lowering the threshold to initiate the response to heat stress. Heat stress increased H_2_O_2_ contents in the red alga *Pyropia tenera*, a response that was attenuated in a *P. tenera* mutant with increased heat stress tolerance (Lee and Choi, 2019). These findings support the tight relationship between reduced H_2_O_2_ accumulation and the acquisition of heat stress tolerance with low sensitivity to heat stress, although the contribution of heat stress memory to the acquisition of heat stress tolerance in *P. tenera* has yet to be analyzed.

In addition, the heat stress memory–dependent inhibition of H_2_O_2_ accumulation at the lethal temperature suggests that less H_2_O_2_ is available to act as a second messenger to activate heat stress signal transduction pathways (Sachdev et al., 2021; Fortunato et al., 2023), which may be related to the modulation of nitrogen transporter gene expression by heat stress memory. Based on this idea, we propose that heat stress memory–dependent inhibition of H_2_O_2_ production reduces the expression and activation of ROS scavengers, such as superoxide oxidase (SOD), catalase (CAT), and ascorbate peroxidase (APX) (Shah and Nahakpam, 2012; Hasanuzzaman et al., 2020; Tiwari et al., 2022). If so, this may underlie the reduced sensitivity of algae to heat stress at lethal temperature in response to heat stress memory; however, this notion remains to be confirmed.

This is the first report of nitrogen transporter genes in the genus *Bangia*. We identified five paralogs of *BE1MAT1* that were phylogenetically divided into five subclades, each of which contained the corresponding orthologs from *P. yezoensis* and *Po. umbilicalis*. These findings suggest that an ancient Bangiales *AMT* gene might have diversified into five genes prior to the separation of *Pyropia*, *Porphyra*, and *Bangia*. In addition, we identified two paralogs, *BE1DUR3.1* and *BE1DUR3.2*, belonging to different red algal DUR3 subclades. Since these subclades contained orthologs from *G. chorda*, *C. crispus*, *Po. umbilicalis*, and *P. yezoensis*, we suggest that the ancestral *DUR3* gene might have been present prior to the separation of Bangiophyceae and Florideophyceae. Moreover, in contrast to AMT1s and DUR3s, multicellular Bangiales contain only one *NRT2* gene, although multiple *NRT2* genes have been identified in unicellular red algae and terrestrial plants (**Figure 3**). These observations suggest that the ancestral *NRT2* gene did not undergo duplication in ancient multicellular algae, but rather diversified in terrestrial plants after their colonization of land.

It is plausible that the differences in these genes between algae and terrestrial plants are due to the differences in their living environments. Since seawater contains high levels of nitrate ion, it appears that the functional diversity of the *NRT2* gene is not required in marine algae. The situation is different in soil, where the production of nitrate ions completely depends on the assimilation of nitrogen gas by bacteria to enable the absorption of nitrate ions by plants (Bekele and Yilma, 2021; Guo et al., 2023). Fresh water also contains lower levels of nitrate ions than seawater. This appears to be responsible for the duplication of the *NRT2* gene in freshwater algae such as *Porphyridium purpureum* and *C. reinhardtii*, which likely contributed to the efficient absorption of low concentrations of nitrogen sources by these algae. By contrast, the requirement for ammonium ions and urea is usually acute in seawater; thus, the multiplication of AMT1 and DUR3 genes might have been necessary for the effective absorption of these molecules by marine algae. Whether there is a relationship between nitrogen transporter gene amplification and the availability of different nitrogen sources in the environment should be verified.

We also addressed whether heat stress and heat stress memory affect the expression levels of nitrogen transporter genes in ‘*Bangia*’ sp. ESS1. We detected the heat stress–inducible expression of *BE1AMT1.3*, *BE1AMT1.5*, and *BE1AMT1.7*, suggesting that these genes contribute to the early phase of the heat stress response. Of these genes, the expression levels of *BE1AMT1.3* and *BE1AMT1.5* were reduced if exposure to the lethal temperature came after priming and recovery, pointing to the reduced sensitivity to heat stress–dependent expression due to heat stress memory.

Although this expression pattern appears to be consistent with the production of H_2_O_2_, as shown in **Figure 1**, the effects of heat stress memory on gene expression profiles were complex. For instance, triggering-dependent expression was observed for *BE1AMT1.4* and *BE1DUR3.1*, whose expression was not induced by priming or direct transfer to the lethal temperature, suggesting that heat stress memory increases the sensitivity of these genes to heat stress. A similar pattern was observed for *BE1AMT1.7*, although priming also induced its expression. In addition, *BE1AMT1.1* showed recovery-dependent and priming- and triggering-independent expression, and triggering highly reduced *BE1NRT2* and *BE1DUR3.2* expression to the levels observed in response to direct transfer to the lethal temperature. These results suggest that the latter two genes are not involved in nitrogen transport under heat stress conditions.

In conclusion, our results indicate that reducing the sensitivity to heat stress by heat stress memory influences mRNA expression in a gene-specific manner. The priming-inducible expression of *BE1AMT1.4* and *BE1AMT1.5* and triggering-dependent expression of *BE1AMT1.4* and *BE1DUR3.1* strongly point to functional diversity in the timing of nitrogen absorption during various phases of heat stress. Therefore, we propose that the main effects of heat stress memory are to reduce priming-dependent gene expression by triggering and to induce the trigger-dependent expression of priming-independent genes. These effects appear to underlie the reduced sensitivity of ‘*Bangia*’ sp. ESS1 to heat stress via the memorization of stress, with differential contributions of nitrogen transporters to the maintenance of nitrogen absorption under heat stress conditions.

We previously demonstrated that ‘*Bangia*’ sp. ESS1 maintains increased levels of various saturated and monounsaturated fatty acids and decreased levels of various polyunsaturated fatty acids as a physiological basis for heat stress memory (Kishimoto et al., 2019). Perhaps the physical state of the membrane, which depends on fatty acid composition, modulates the activities of membrane-bound nitrogen transporters, which might control the expression patterns of the genes encoding them via a feedback mechanism. Thus, both the activities of nitrogen transporters in membranes in various physical states and the mechanisms regulating nitrogen transporter gene expression under heat stress conditions must be elucidated. Notably, the capability to establish heat stress memory varies among species in the genus *Bangia* (Khoa et al., 2021). To understand why heat stress response strategies vary among species, comparative analyses of heat stress–dependent H_2_O_2_ production and the expression profiles of nitrogen transporter genes should be performed using ‘*Bangia*’ sp. ESS2, which lacks heat stress memory. These approaches could further our understanding of the relationship between heat stress memory and growth regulation for the survival of Bangiales under unfavorable high-temperature conditions.

## 5 Conclusion

In this study, we addressed the biological significance of heat stress memory in the red alga ‘*Bangia*’ sp. ESS1. By comparing changes in the quantities of H_2_O_2_ and transcripts from genes encoding nitrogen transporters such as BE1AMT1s, BE1NRT2, and BE1DUR3s, we identified priming- and triggering-dependent sets of nitrogen transporter genes whose expression is repressed and promoted, respectively, by heat stress memory. These different expression profiles promote heat stress tolerance and protect the organism by maintaining nitrogen absorption at high temperatures. Our study provides unique insights into the strategies of heat stress responses and adaptation via the establishment of heat stress memory in aquatic photosynthetic organisms.

## Data availability statement

The amino acid sequence data presented in this study can be found in online repositories. The names of the repository/repositories and accession number(s) can be found in the article. The original contributions presented in the study are included in the article/**Supplementary Material**. Further inquiries can be directed to the corresponding author.

## Author contributions

NS: Data curation, Formal analysis, Investigation, Methodology, Validation, Visualization, Writing – review & editing. HK: Data curation, Formal analysis, Investigation, Methodology, Validation, Visualization, Writing – review & editing. KM: Conceptualization, Data curation, Formal analysis, Funding acquisition, Investigation, Methodology, Supervision, Validation, Visualization, Writing – original draft, Writing – review & editing.
