# Supplementary material for: Heat stress memory differentially regulates the expression of nitrogen transporter genes in the filamentous red alga ‘Bangia’ sp. ESS1
Source: Front Plant Sci. 2024 Feb 5;15:1331496. doi: 10.3389/fpls.2024.1331496 (PMC10875135; doi:10.3389/fpls.2024.1331496)
Supplement: Supplementary file 1 [file DataSheet_1.pdf]

Supplementary Table 1. Sequences of primers used for qRT-PCR

Primer name	Sequence (5'-3')	Product size (bp)
Q-BE1AMT1.1-F	GCGGAGCAAGAACACGAAGA	94
Q-BE1AMT1.1-R	GGCAAAGGCATACCCAAACAG	
Q-BE1AMT1.3-F	TGGCAACGAGGGAAATGG	85
Q-BE1AMT1.3-R	GAAGAAGAATGCGAGGGAGTG	
Q-BE1AMT1.4-F	GCGGGCAAAAAAACACCAAGA	96
Q-BE1AMT1.4-R	GGGCAAACGCATTCCCAAAC	
Q-BE1AMT1.5-F	CATTTACCAGCTCCCATTCCC	199
Q-BE1AMT1.5-R	CCATAGCCCAACCACCCATACT	
Q-BE1AMT1.6-F	CCCCACCATAAACCCCTT	111
Q-BE1AMT1.6-R	TGCTGCCCCGTTACCATC	
Q-BE1Rh-F	CCTACTTTTTCCTTGTCTTTCA	102
Q-BE1Rh-R	CACCATTATCTACCGCTTTATC	
Q-BE1NRT2-F	CACTTTGCGTGGTCGTCCTT	108
Q-BE1NRT2-R	CGCCGAGTCATCGTTCAGC	
Q-BE1DUR3.1-F	AGGAAGGGGTCAAGGCTGTT	182
Q-BE1DUR3.1-R	CGCATTCGGGTTGGGTGTA	
Q-BE1DUR3.2-F	GCGTTTGGGATCTCCTTTGG	128
Q-BE1DUR3.2-R	GCGGTGAGCCCCGTCTTG	
Q-BEActin-F	TCAACCCCAAGGCCAACC	150
Q-BEActin-R	TCACGCCGTCCCCAGAAT	
