# Supplementary figures and images for: Heat stress memory differentially regulates the expression of nitrogen transporter genes in the filamentous red alga ‘Bangia’ sp. ESS1
Source: Front Plant Sci. 2024 Feb 5;15:1331496. doi: 10.3389/fpls.2024.1331496 (PMC10875135; doi:10.3389/fpls.2024.1331496)

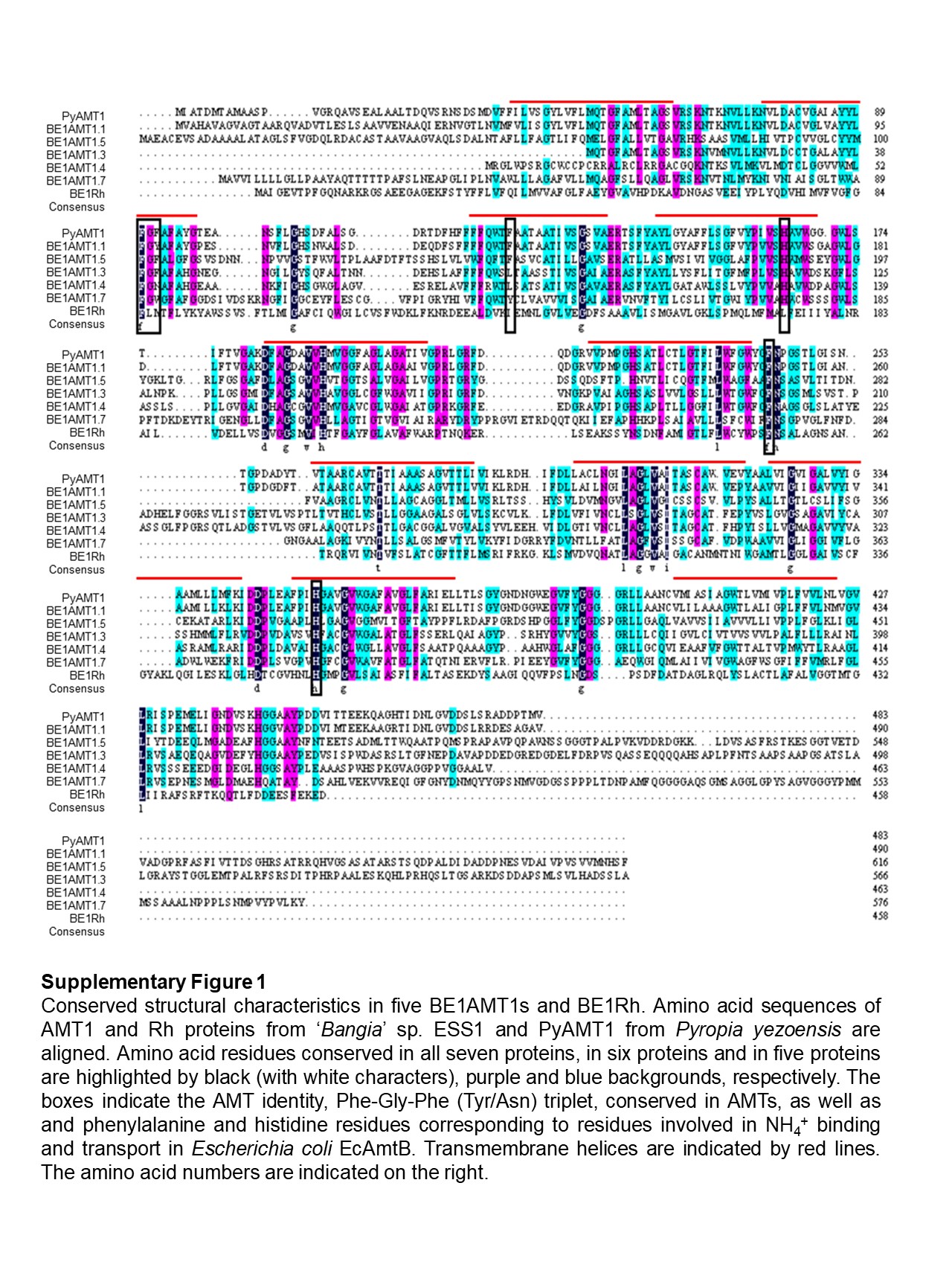

Supplement: Supplementary file 2 [file Image_1.jpeg]

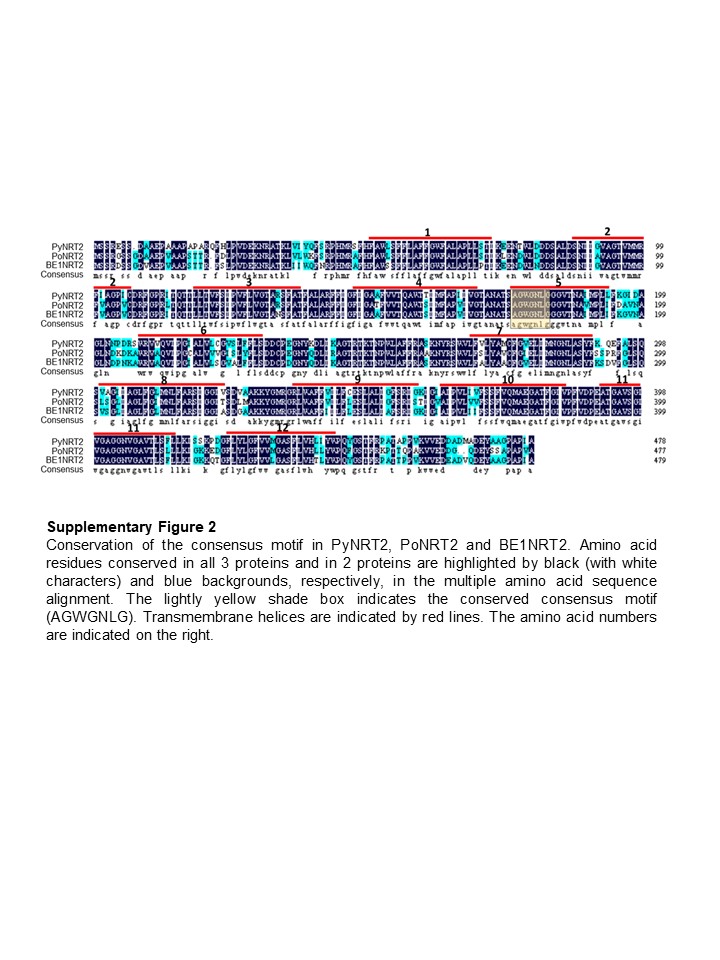

Supplement: Supplementary file 3 [file Image_2.jpeg]

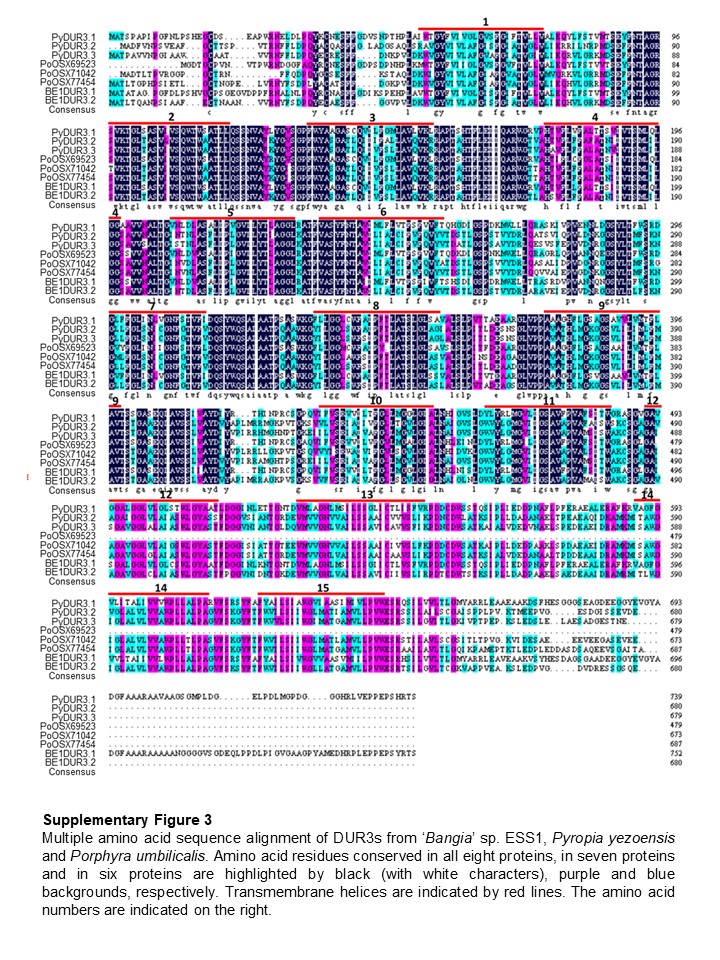

Supplement: Supplementary file 4 [file Image_3.jpeg]
